# Coping-Strategies as a Mediator Between Symptoms of Sleeping Problems and Risk of Suicide

**DOI:** 10.62641/aep.2384

**Published:** 2026-10-15

**Authors:** Celia Antuña-Camblor, Óscar Peris-Baquero, Héctor Morillo-Sarto, Joel Juarros-Basterretxea

**Affiliations:** ^1^Faculty of Psychology and Health Sciences, Open University of Madrid (UDIMA), 28400 Collado Villalba, Madrid, Spain; ^2^Department of Psychology and Sociology, University of Zaragoza, 44003 Teruel, Aragón, Spain; ^3^Health Research Institute of Aragon (IIS Aragon), 50009 Zaragoza, Aragón, Spain; ^4^Department of Psychology and Sociology, University of Zaragoza, 50009 Zaragoza, Aragón, Spain

**Keywords:** sleep problem, coping skills, suicidal risk, mediation analysis, emotion regulation

## Abstract

**Background:**

Suicide is a complex, global phenomenon linked to various intersecting vulnerability factors. Among these, sleep-related symptoms represent a significant yet understudied contributor to suicide risk. This study explored whether the concurrent relationship between sleep-related symptoms and suicide risk is statistically mediated by distinct coping strategies within an exploratory cross-sectional framework, addressing data non-normality through robust analytical methods.

**Methods:**

A total of 1014 participants (33.83% male, 66.17% female) aged 18 to 75 years (M = 33.00, SD = 15.15) participated in an online survey distributed through snowball recruitment. Participants completed the amplified Sleep Symptom Subscale (Su-a) of the Brief Symptom Checklist (LSB-50), the Coping with Stress Questionnaire (CSQ), and the Risk of Suicide Scale (RS). Multiple mediation analyses were conducted to evaluate the indirect effects of coping dimensions while adjusting for sex, age, educational attainment, and socioeconomic status. Results: Sleep-related symptoms were positively associated with suicide risk. Negative self-focus emerged as the strongest mediator (β = 0.112, 95% CI [0.083, 0.143]), whereas Positive Reappraisal (β = 0.018, 95% CI [0.007, 0.032]) and Seeking Social Support (β = 0.012, 95% CI [0.001, 0.024]) acted as protective mediators. Overt emotional expression also showed a small but significant indirect effect (β = 0.009, 95% CI [0.001, 0.018]). A significant direct effect of sleep-related symptoms on suicide risk remained, indicating partial mediation.

**Conclusions:**

The findings suggest that coping processes contribute to the connection between sleep difficulties and suicide risk. Interventions aimed at reducing maladaptive coping and strengthening adaptive coping skills may help diminish suicidal vulnerability among individuals experiencing sleep disturbances.

## Introduction

Suicide is a global and complex phenomenon with a multitude of social, family, and health consequences [1]. According to the 2019 report of the World Health Organization (WHO), it is estimated to claim approximately 720,000 lives per year [1]. In Spain, the figures are also alarming and continue to grow year after year. For example, a nationwide registry study found that suicide rates in Spain increased from 8.4 to 9.3 per 100,000 between 2018 and 2023, with an average annual increase of 2.6% [2] that is why, to reduce this rate, an attempt has been made to study what factors affect suicide risk.

Research has predominantly focused on individual aspects related to suicidal behavior and has identified several sociodemographic and psychological factors that influence suicide risk. Starting with the sociodemographic data, in terms of age, the average age of adulthood stands out. Thus, data indicates midlife (30–59 years) represent high rates [3]. In terms of gender, the majority was men [2, 4, 5]. Another sociodemographic variable that has proven relevant to suicide rates is socioeconomic status. In this regard, the highest suicide rates have been found in lower socioeconomic levels [6, 7]. Psychological risk factors have also been found, such as rumination [8, 9] or intolerance of distress [10]. It has also been linked to mental health [11] such as emotional disorders [9], or substance use disorders [12, 13]. However, not so much importance has been given to a problem that appears frequently in diagnoses, such as sleep disturbances [14, 15].

Sleep disturbances are any condition that disrupts the normal sleep pattern, making it difficult or even impossible to get restorative sleep and encompass insomnias, hypersomnia, disorders of the sleep-wake schedule, and parasomnias [16]. These disturbances are highly prevalent in the general population [17, 18], deteriorating the psychological well-being and mental health of those who suffer from it [19].

In the context of the Integrated Motivational-Volitional Model (IMV) of suicidal behavior [20, 21], sleep problems are set in the pre-motivational phase, considered as a background vulnerability that acts as a major cognitive stressor. The bidirectional relation between sleep disturbances and mental health deterioration is well established [20, 21]; on the one hand, mental health concerns cause sleep disturbances. On the other hand, it is also known that sleep disturbances frequently lead to the appearance, recurrence, and maintenance of mental health concerns [19, 22]. Constant feedback between mental health deterioration and sleep disturbances reinforces a pattern of psychological suffering and distress (i.e., anxiety and depression) and impairs a person’s ability to cope with daily life, which in turn increases the risk of suicide [15, 23].

According to the IMV model [20, 21], the formation of suicidal ideation occurs during the motivational phase. During this phase, the individuals develop suicidal distress characterized by the experience of feeling defeated and the pervasive sense of entrapment, leaving the individual without any apparent means of adaptively changing the situation [24]. In this context, the ultimate form of experiential avoidance, suicidal behavior, appears as a valid alternative to escape for unbearable suffering [25, 26]. The transition from sleep problems to suicidal distress and subsequent suicidal behavior is affected by different factors, such as coping strategies [27, 28]. While those individuals with adaptive coping strategies tend not to endorse experiential avoidance, the likelihood to endorse maladaptive suicidal and suicide related behaviors increases when lacking them [29, 30, 31]. For example, Otsuka *et al*. [32] discovered how people with sleep disorders are more likely to resort to maladaptive coping strategies, which can exacerbate sleep problems and increase vulnerability to stress. Conversely, adaptive coping strategies can reduce the negative impact of sleep disorders, potentially protect mental health, and reduce suicide risk. These findings support the idea that coping strategies may mediate the relationship between sleep disorders and suicide risk, highlighting the importance of promoting adaptive coping to buffer the effects of insomnia. In addition, Roberge *et al*. [33] discovered that changes in cognitive flexibility predicted changes in sleep disorder symptoms and that improving cognitive flexibility improved both sleep problems and suicide risk.

For these reasons, and due to the high prevalence of suicide risk, it is crucial to continue exploring the relationship between these variables and how they influence each other to reduce suicide risk and its prevalence. Building on prior findings, this study addresses a significant omission in current research. To the best of our knowledge, no existing investigation has concurrently analyzed the full spectrum of coping mechanisms as a mediating factor between sleep disturbances and suicidal risk, while adjusting for sociodemographic factors (age, sex, education, and socioeconomic status) within a Spanish adult sample. Based on the theoretical landscape, we hypothesized that adaptive strategies such as seeking social support and positive reappraisal would act as protective pathways, attenuating the concurrent association between sleep problems and suicide risk. Conversely, we expected that maladaptive coping strategies such as negative self-focus would operate as risk factors, increasing the vulnerability to suicide risk.

## Methods

### Sample and Procedure

Data were gathered between August 2022 and September 2023 using an online cross-sectional design. Recruitment was conducted through a snowball dissemination strategy in which participants were invited to complete the survey and share the study link with individuals in their social networks. Participation was entirely uncompensated and voluntary, requiring roughly 30 minutes to complete. Because of the study’s observational framework, we applied no exclusion criteria other than being underage (under 18). Ethical oversight and research integrity were maintained via approval from the Research Ethics Committee of the Principality of Asturias under approval number 2022.193. According to the Declaration of Helsinki, prior to participation, all respondents were provided with detailed information regarding the study objectives, procedures, confidentiality safeguards, and their rights as participants. Electronic informed consent was obtained from all participants before access to the survey was granted. Participation was anonymous, and participants were informed that they could discontinue the survey at any time without penalty. All procedures were conducted in accordance with the ethical principles outlined in the Declaration of Helsinki and its subsequent amendments.

A total of 1763 individuals initially accessed the survey link. Of these, 749 records were excluded because they represented early dropouts (empty records and completing only the sociodemographic block), without providing any data on the core scales. Consequently, the final analytical sample comprised 1014 participants who completed the entire protocol. The participants were aged 18 to 75 (M = 33.0, SD = 15.15), of whom 66.17% identified as female and 33.83% as male. Further sample characteristics are outlined in Table 1.

**Table 1.** **Sociodemographic characteristics of the sample**.

		Sample	
Sociodemographic variables		(n = 1014)	
		n	%
Sex			
	Male	343	33.83
	Female	671	66.17
Age			
	18–25 years	546	53.85
	26–39 years	164	16.17
	40–59 years	265	26.13
	60+ years	39	3.85
Marital status			
	Single	343	33.83
	Married	312	30.77
	Divorced	183	18.05
	Unmarried couple	161	15.88
	Widower	15	1.48
Educational level			
	Basic education	184	18.15
	Secondary education	184	18.15
	Baccalaureate studies	423	41.72
	University studies	223	21.99
Socioeconomic status			
	Low	136	13.41
	Medium	618	60.95
	Medium-high	254	25.05
	High	6	0.59
Family history of mental health			
	Yes	190	18.74
	No	824	81.26

### Instruments

Ad-hoc sociodemographic questionnaire. A custom ad-hoc questionnaire was designed to capture the demographic profile of the cohort. This instrument assessed age, marital status, family psychiatric history, educational attainment, and socioeconomic status via single-item indicators. Although sex and gender were initially surveyed separately, they were merged into a single category due to an absence of empirical differences between them in our dataset. Notably, 54.1% of the respondents reported undergoing concurrent psychological treatment during the data collection period. All sociodemographic variables were self-reported by the participants through the online survey.

Sleep subscale of the Brief Symptom Checklist (LSB-50) [34], which is a validated and recognized tool in psychological research. The amplified Sleep Symptom Subscale (Su-a) is composed of seven items to assess the specific presence of sleep disturbances alongside manifestations from anxiety and depression. Participants rated the frequency or intensity of each symptom on a five-point scale ranging from 0 (“not at all”) to 4 (“extremely”). Higher scores reflected greater impairment associated with sleep problems. In the current sample, the scale demonstrated satisfactory internal consistency (Cronbach’s α = 0.84).

The Coping with Stress Questionnaire (CSQ) [35] is a 42-item self-report instrument designed to assess different ways individuals manage stressful situations. The questionnaire evaluates seven coping dimensions with six items each: Problem-solving focus (PSF; α = 0.82), which reflects active efforts to address the source of stress; Positive reappraisal (PRE; α = 0.80), involving reinterpretation of stressful experiences more constructively; Seeking social support (SSS; α = 0.85), referring to obtaining emotional or practical assistance from others; Negative self-focus (NAF; α = 0.86), characterized by self-blame, pessimism, and feelings of helplessness; Overt emotional expression (OEE; α = 0.74), reflecting the overt expression of emotions generated by stressful events; Avoidance (AVO; α = 0.75), which involves attempts to disengage from or suppress stress-related thoughts; and Religion (REL; α = 0.88), referring to the use of spiritual or religious practices as a coping resource. Higher scores indicate more frequent use of the corresponding coping strategy more constructively.

Risk of Suicide Scale (RS) [36, 37]. This instrument consists of 15 dichotomous items answered with “yes” or “no”. Affirmative responses are summed to obtain a total score, with higher scores indicating greater vulnerability to suicidal behavior. Owing to the anonymous nature of data collection, it was not possible to identify participants whose scores exceeded established risk thresholds. Nevertheless, all participants were provided with information about available mental health and crisis support services upon completion of the survey. Internal consistency for the scale in the present sample was good (α = 0.82).

### Analysis

Statistical computations were carried out using IBM-SPSS (v.27; IBM Corp., Armonk, NY, USA) [38] and R (v4.3.3; R Foundation for Statistical Computing, Vienna, Austria) [39]. Initial descriptive statistics characterized the sample distributions, and collinearity diagnostics confirmed that multicollinearity assumptions were not violated. To prevent potential biases in standard errors and significance testing resulting from these non-normal and overdispersed parameters, parallel mediation analysis was performed using the PROCESS macro (v5.0) in R. To fully protect the linear equations against heteroscedasticity and violations of normality, Davidson–MacKinnon heteroscedasticity-consistent standard error estimators (HC3) were applied to all paths. Furthermore, all continuous quantitative predictors, mediators, and outcomes were fully standardized before model entry to compute standardized regression coefficients (β).

The multivariate parallel mediation configuration (PROCESS Model 4) defined the LSB-50 Sleep subscale score as the independent variable (X), the suicide risk score as the dependent variable (Y), and the seven coping subscales as concurrent mediators (M1 to M7) (See Fig. 1). Age, sex, educational level, and socioeconomic status were simultaneously integrated into the model as controlled covariates. To formalize the evaluation of the indirect pathways under non-normal conditions, 95% bias-corrected bootstrap confidence intervals (95% BC CI) for all specific and total indirect effects were computed utilizing 5000 resamples. This non-parametric resampling strategy does not impose distributional assumptions, and an indirect effect was deemed statistically significant if its bootstrap confidence interval completely excluded zero.

**Fig. 1.**
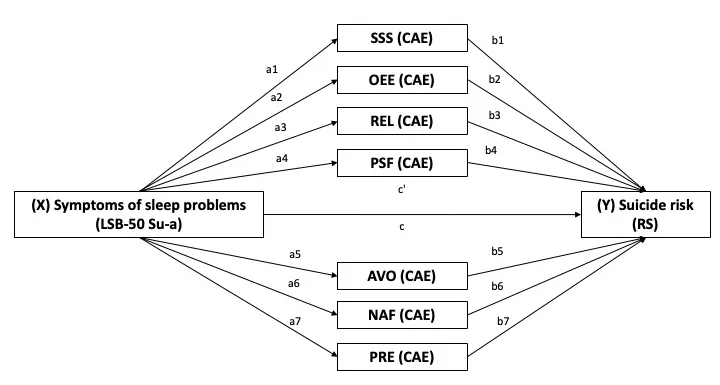
**Hypothetical mediation model**. SSS, seeking social support; OEE, overt emotional expression; REL, religion; PSF, problem-solving focus; AVO, avoidance; NAF, negative self-focus; PRE, positive reappraisal; RS, Risk of Suicide Scale; LSB-50, Brief Symptom Checklist; Su-a, amplified Sleep Symptom Subscale.

To test the plausibility of alternative directional or antecedent pathways within our cross-sectional framework and address potential temporal ambiguity, a reverse mediation sensitivity model was calculated following identical statistical procedures. This configuration inverted the parameters, setting suicide risk as the predictor (X) and sleep disturbances as the primary outcome (Y), while maintaining the same parallel mediators and covariates.

## Results

The sociodemographic distribution of the sample is presented in Table 1. Overall, the sample was predominantly characterized by young, female participants. Regarding the marital status, single and married individuals represented the main cohorts. In terms of educational level, the majority of the sample had completed baccalaureate and university studies. Additionally, most respondents perceived themselves as belonging to a medium socioeconomic status. Finally, the participants who reported a family history of mental health constituted a clear minority.

### Mediation Analysis

First, the descriptive and distribution analysis were carried out (see Table 2). Overall, most variables showed acceptable skewness and kurtosis parameters. None of the variables showed a normal distribution.

**Table 2.** **Descriptive and distribution analyses for independent, dependent, and mediational variables**.

Variable	M	SD	Median [P25, P75]	Skewness	Kurtosis	W
Sleep problems	1.42	0.91	1.14 [0.71, 2]	0.82	–0.08	0.93***
Seeking social support	11.22	6.40	11 [6, 16]	0.20	–0.74	0.97***
Overt emotional expression	6.62	3.74	6 [4, 9]	0.52	–0.22	0.97***
Religion	2.45	4.32	0 [0, 3]	2.15	4.39	0.64***
Problem-solving focus	12.13	5.13	12 [9, 16]	–0.02	–0.45	0.99***
Avoidance	10.62	3.95	11 [8, 13]	0.18	0.34	0.99***
Negative self-focus	8.59	4.29	8 [6, 11]	0.54	0.42	0.98***
Positive reappraisal	12.72	4.72	13 [10, 16]	–0.05	–0.14	0.99***
Suicide risk	4.42	3.38	4 [2, 7]	0.68	–0.31	0.93***

Note. M, mean; SD, standard deviation; W, Shapiro–Wilk test; ***p < 0.001.

As shown in Table 3, sleep problems were significantly associated with almost all coping strategies. Specifically, higher sleep problems were associated with a significant decrease in adaptive protective strategies, such as SSS, PSF, and PRE. Conversely, sleep problems were positively associated with risk-amplifying strategies, including OEE, AVO, and NAF. No significant link was found between sleep symptoms and REL.

**Table 3.** **Direct, indirect, and total effects of the model**.

Model path			β [95% CI]	SE
Direct effects				
	Paths a (IV → Mediator)			
		Sleep problems → Seeking social support	–0.207 [–0.269,–0.145]	0.031
		Sleep problems → Overt emotional expression	0.152 [0.092, 0.212]	0.031
		Sleep problems → Religion	0.010 [–0.056,0.068]	0.031
		Sleep problems → Problem-solving focus	–0.132 [–0.196, –0.068]	0.033
		Sleep problems → Avoidance	0.108 [0.040, 0.176]	0.035
		Sleep problems → Negative self-focus	0.478 [0.419, 0.537]	0.030
		Sleep problems → Positive reappraisal	–0.156 [–0.221, –0.091]	0.033
	Paths b (Mediator → DV)			
		Seeking social support → Suicide risk	–0.057 [–0.111, –0.002]	0.028
		Overt emotional expression → Suicide risk	0.056 [–0.001, 0.112]	0.029
		Religion → Suicide risk	–0.066 [–0.116, –0.016]	0.026
		Problem-solving focus → Suicide risk	0.024 [–0.034, 0.082]	0.030
		Avoidance → Suicide risk	–0.023 [–0.073, 0.028]	0.026
		Negative self-focus → Suicide risk	0.235 [0.173, 0.296]	0.031
		Positive reappraisal → Suicide risk	–0.117 [–0.180, –0.054]	0.032
	Path c′ (IV → DV)			
		Sleep problems → Suicide risk	0.511 [0.458, 0.564]	0.027
Indirect effects				
	Sleep problems→ Suicide risk			
		Via seeking social support	0.012 [ 0.001, 0.024]	0.006
		Via overt emotional expression	0.009 [0.001, 0.018]	0.005
		Via religion	–0.001 [–0.009, 0.001]	0.002
		Via problem-solving focus	–0.003 [–0.010, 0.010]	0.004
		Via avoidance	–0.003 [–0.010, 0.003]	0.003
		Via negative self-focus	0.112 [0.083, 0.143]	0.015
		Via positive reappraisal	0.018 [0.007, 0.032]	0.006
	Total indirect		0.145 [0.112, 0.177]	0.017
Total effect			0.664 [0.610, 0.701]	0.023

Note: β, standardized regression coefficient; CI, confidence interval; IV, independent variable; DV, dependent variable; SE, standard error. Confidence intervals for indirect effects were estimated using 5000 bootstrap samples. Models were adjusted for age, sex, educational level, and socioeconomic status.

Regarding the direct effects on the primary outcome (suicide risk), several coping mechanisms emerged as significant predictors while adjusting for sleep symptoms and covariates. NAF was positively associated with suicide risk, whereas OEE exhibited a marginally non-significant positive relationship. Conversely, PRE, REL, and SSS were significantly and negatively associated with suicide risk. PSF and AVO did not exhibit direct statistical associations with suicide risk. Crucially, sleep problems retained a powerful, positive, and highly significant direct effect on suicide risk even after fully adjusting for all simultaneous mediators and covariates. The persistence of a significant direct effect (β = 0.511, *p* < 0.001) indicates partial mediation, suggesting that coping strategies explain only part of the association between sleep problems and suicide risk.

The evaluation of the specific indirect effects revealed four statistically significant mediation pathways. NAF emerged as the strongest mediator of the association between sleep problems and suicide risk (β = 0.112, 95% CI [0.083, 0.143]). PRE also showed a significant indirect effect (β = 0.018, 95% CI [0.007, 0.032]), as did SSS (β = 0.012, 95% CI [0.001, 0.024]) and OEE (β = 0.009, 95% CI [0.001, 0.018]). In contrast, the indirect effects through REL (β = −0.001, 95% CI [−0.009, 0.001]), PSF (β = −0.003, 95% CI [−0.010, 0.010]), and AVO (β = −0.003, 95% CI [−0.010, 0.003]) were not statistically significant because their bootstrap confidence intervals included zero. The total indirect effect was statistically significant (β = 0.145, 95% CI [0.112, 0.177]).

To systematically address the causal directionality limitations and temporal ambiguity inherent to the cross-sectional design, a comprehensive reverse sensitivity mediation model was computed. When inverting the parameters—modeling suicide risk as the independent variable (X) and sleep disturbances as the primary outcome (Y)—the statistical behavior changed significantly.

The reverse sensitivity analysis yielded a non-statistically significant total indirect effect (β = 0.094, 95% CI [–0.080, 0.150]). Significant indirect effects were observed through NAF (β = 0.100, 95% CI [0.060, 0.130]), whereas the remaining indirect pathways were not statistically significant (Table 4). Nevertheless, because all variables were assessed concurrently, neither model can establish temporal precedence or causal directionality.

**Table 4.** **Indirect effects of suicide risk on sleep problems (sensitivity analysis)**.

Indirect effects		β [95% CI]	SE
Suicide risk → Sleep problems			
	Via seeking social support	0.003 [–0.010, 0.030]	0.010
	Via overt emotional expression	–0.000 [–0.010, 0.010]	0.010
	Via religion	–0.001 [–0.010, 0.010]	0.000
	Via problem-solving focus	–0.001 [–0.010, 0.010]	0.001
	Via avoidance	0.001 [–0.001, 0.010]	0.000
	Via negative self-focus	0.100 [0.060, 0.130]	0.020
	Via positive reappraisal	–0.009 [–0.029, 0.010]	0.010
	Total indirect	0.094 [–0.080, 0.150]	0.020

Note. β represents bootstrapped standardized coefficients. SE corresponds to the standard error estimated via bootstrapping. 95% Bootstrap CI represents the bias-corrected percentile confidence interval based on 5000 resamples. CI, confidence interval; SE, standard error.

## Discussion

By controlling for sociodemographic factors (age, sex, socio-economic status, and educational level), this study examines how various coping mechanisms mediate the link between sleep problems and suicide risk. Our findings confirm a partial mediation effect where different strategies play opposing roles. This partial mediation supports the hypothesis of coping strategies affecting the accounting for the concurrent pathways through which background vulnerabilities, such as sleep problems, connect with suicidal risk; at the same time, it indicates that coping strategies do not capture the full extent of the effect of these vulnerabilities, emphasizing the necessity to take into account additional threatening to self and motivational aspects [20, 21].

Four coping strategies showed statistically significant indirect effects: SSS, PRE, OEE, and NAF. However, the strongest mediation effect was observed for NAF, whereas the effects through SSS, PRE, and OEE were comparatively modest. These results indicate that although adaptive coping strategies may be negatively associated with suicide risk, they are also negatively affected by sleep problems. In other words, results suggest that those individuals suffering sleep problems may struggle to adaptively cope with daily life events, increasing the likelihood of suicide related behaviors [15, 22] and thus, adaptive coping strategies play a key, yet vulnerable, protective role.

Regarding the social support-seeking coping strategy, our results indicate that sleep problems are associated with a reduction in seeking social support, which in turn is associated with higher suicide risk. The indirect effect through SSS was statistically significant (β = 0.012), indicating that sleep problems may erode individuals’ tendency to seek support from others. This finding is consistent with evidence suggesting that sleep disturbances impair interpersonal functioning, increase irritability, and foster withdrawal from social relationships. Consequently, individuals experiencing sleep problems may become progressively disconnected from protective social resources, thereby increasing vulnerability to suicidal thoughts and behaviors. This aligns with previous research indicating the relevance of social support to prevent suicide but also emphasizes the pernicious effect of sleeping problems on social network maintenance and development. Previous research has demonstrated that background vulnerabilities such as sleep problems increase irritability and cognitive biases that negatively affect interpersonal interactions, pushing significant others away and withdrawing from the social environment, increasing isolation and decreasing help-seeking behaviors, increasing the likelihood of maladaptive coping such as suicide [31]. In terms of intervention, these results suggest that maintenance and development of robust social support networks can be a key element to prevent the evolution of sleep problems in suicide related behaviors.

Similarly, our findings support the protective role of PRE. Sleep problems were associated with lower levels of PRE (β = −0.156), whereas PRE was associated with lower suicide risk (β = −0.117). Consequently, reductions in the use of this coping strategy partially explained the relationship between sleep problems and suicide risk. PRE facilitates cognitive flexibility, emotional regulation, and the ability to reinterpret adverse situations in a less threatening manner. Therefore, when sleep disturbances compromise these cognitive capacities, individuals may become less able to generate alternative interpretations of stressful experiences, increasing feelings of hopelessness and psychological pain [40].

Regarding maladaptive coping strategies, the results revealed a more nuanced pattern. NAF showed a robust positive association with suicide risk, whereas OEE displayed only a weak association and AVO was not significantly associated with suicide risk after controlling for the remaining coping dimensions. Nevertheless, significant indirect effects were observed for OEE and NAF, indicating that sleep problems may increase suicide risk through these pathways. Nevertheless, sleep problems only showed a statistically significant indirect and positive effect on suicide risk through OEE and NAF. Contrary to adaptive coping, the positive indirect effect was due to consecutive positive associations; individuals’ sleep problems were associated with higher use of OEE and NAF, which in turn was associated with higher suicide risk.

Although the indirect effect through OEE reached statistical significance, its magnitude was relatively small (β = 0.009), and the direct association between OEE and suicide risk was marginal (*p* = 0.051). Therefore, this pathway should be interpreted more cautiously than the substantially stronger mediation observed through NAF. As sleep problems consume individuals’ cognitive and emotional capacity, they are more prone to emotional dysregulation and more likely to engage in unmodulated, eternalized, and volatile emotional expression [35]. This overt expression fails to resolve the underlying tension and exacerbates interpersonal conflicts, negatively affecting the immediate social support network, and accelerates emotional crisis. In combination, the OEE generates a climate of increased entrapment and loneliness that increases the likelihood of displaying maladaptive responses, such as suicide. In terms of intervention these findings highlight the need for emotional regulation training and interpersonal skills programs that teach individuals how to modulate and communicate their distress constructively. By equipping individuals with strategies to manage acute affective storms—such as de-escalation techniques or distress tolerance skills—clinicians can help minimize interpersonal friction, preserve vital social support networks, and disrupt the escalation from emotional volatility to acute suicidal crises.

Most importantly, NAF emerged as the strongest mediating pathway in the entire model. Sleep problems showed a strong positive association with NAF (β = 0.478), and NAF was, in turn, strongly associated with suicide risk (β = 0.235). This resulted in the largest indirect effect observed (β = 0.112), accounting for most of the total mediation effect. While OEE represents the projection of distress onto others, NAF turns distress inward, internalizing the stressors [41]. In this regard, this finding is coherent with previous research linking self-critical and ruminative thought patterns to increased risk for suicidal ideation and behaviors [8, 9, 42]. Sleep problems usually leave individuals trapped in prolonged periods of isolation and ruminative loops, increasing the likelihood of helplessness. In terms of intervention, it has been shown that cognitive-behavioral therapies targeting suicide risk yield significant improvements in sleep quality as one of the consequences of addressing transdiagnostic elements to sleep problems and suicidal behaviors, such as rumination [42, 43, 44]. By actively intervening on NAF as the primary target that traps individuals can effectively dismantle the psychological bridge between sleep problems and suicide. These findings suggest that self-critical, self-blaming, and ruminative cognitive processes may constitute one of the principal psychological mechanisms linking sleep disturbances to suicidal vulnerability. Individuals experiencing persistent sleep problems may become increasingly trapped in cycles of negative self-evaluation and repetitive maladaptive thinking, thereby intensifying hopelessness and perceived inability to cope with distress.

An additional finding deserving attention concerns Religious Coping. An additional finding deserving attention concerns Religious Coping. Although this coping dimension was significantly associated with lower suicide risk (β = −0.066), it did not function as a significant mediator because sleep problems were not associated with Religious Coping (β = 0.010, *p* = 0.847). This pattern suggests that Religious Coping may be associated with lower suicidal vulnerability independently of sleep-related symptoms, rather than representing a mechanism through which sleep problems are linked to suicide risk. Future studies should further examine whether religious beliefs and practices are related to suicide risk through pathways not directly connected to sleep disturbances.

Despite the relevance of our results, certain limitations warrant caution when interpreting the data. Specifically, the cross-sectional nature of the study precludes the determination of definitive temporal sequences. Since all measurements were taken concurrently, we cannot empirically verify the chronological order of the observed phenomena. Although a reverse sensitivity model was estimated, neither specification can establish temporal ordering because all variables were assessed simultaneously. However, our statistical approach, parallel mediation design with robust estimators (PROCESS Model 4, HC3), allowed us to map these pathways while controlling data overdispersion. Secondly, the evaluation was in a self-administered format, which can lead to several biases, such as social desirability. Third, the non-homogeneous nature of the sample warrants caution, given that variables like age, educational level, socioeconomic status, and sex heavily weigh on suicide risk profiles. Specifically, our sample is heavily skewed toward young, highly educated, and female participants of medium socioeconomic status. Consequently, these findings cannot be readily generalized to the broader Spanish population, particularly to older demographics, individuals with lower educational attainment, or males, who statistically exhibit the highest rates of completed suicide. We sought to mitigate this issue by first testing a moderated mediation model where sex served as the moderator, though the resulting interaction indices were non-significant (see **Supplementary Table 1**). We therefore included these key demographics as control variables in our main analyses. Nevertheless, we encourage future studies to employ formal invariance testing to confirm that these relationships hold across diverse sociodemographic groups. Finally, a composite measure of suicidal risk was used in the current research. Despite being the most common approach in suicidology, recent contributions in the field suggest that analyzing specific suicidal behaviors as distinct but interrelated elements may offer a more nuanced understanding of the suicidal process than aggregate scores alone. Future research should address this limitation by conducting analyses that delve deeper into the specificity of suicide and suicide-related behaviors for more precise knowledge. This is particularly relevant given that individuals may present similar overall risk scores but differ substantially in their specific behavioral patterns.

Regarding methodology, future lines of research should transcend the constraints of cross-sectional designs by embedding longitudinal tracking to clarify the exact directional pathways between variables. To minimize the biases inherent to self-report measures, such studies would benefit from incorporating multi-source data, including behavioral challenges and clinical interviews. Furthermore, extending this research to clinical populations and ensuring a more equitable gender balance will be critical to testing the limits of these models and examining potential subgroup variations.

The present findings suggest that several coping dimensions are statistically associated with the relationship between sleep-related symptoms and suicide risk. By demonstrating that NAF constitutes the most important pathway linking sleep problems with suicide risk, while PRE and SSS function as protective coping mechanisms, the present study provides a more detailed understanding of the psychological processes underlying suicidal vulnerability. Furthermore, the persistence of a substantial direct effect between sleep problems and suicide risk indicates that coping strategies explain only part of this association and that additional psychological and contextual mechanisms remain to be identified. Ultimately, these insights offer a more nuanced understanding of how sleep pathologies translate into acute clinical vulnerability. From both clinical and preventive standpoints, these findings underscore the necessity of addressing emotional literacy in patients with sleep disturbances. Rather than viewing sleep debt and suicidal vulnerability as distinct entities, our results align with the models proposed by Al-Halabí and Fonseca-Pedrero [45]. Their work underscores those suicidal tendencies often stem from interconnected root processes, including poor distress tolerance and maladaptive coping. Consequently, fragmented treatments are insufficient; effective prevention demands multidimensional strategies that address these overlapping domains concurrently.

## Conclusions

This study examined the association between sleep-related symptoms, coping strategies, and suicide risk in a large Spanish adult sample. The findings suggest that the association between sleep problems and suicide risk is only partially explained by coping strategies NAF emerged as the dominant mediating mechanism, accounting for the largest proportion of the indirect association between sleep problems and suicide risk. In contrast, PRE and SSS appeared to operate as protective factors, whereas OEE showed a smaller but significant indirect effect. Religious Coping was directly associated with lower suicide risk, although it did not mediate the relationship between sleep problems and suicidal vulnerability. These findings should be interpreted cautiously because of the cross-sectional nature of the study, which prevents conclusions regarding temporal precedence or causality. Nevertheless, the results highlight coping processes as potentially relevant correlates of the association between sleep-related symptoms and suicidal vulnerability. Longitudinal and prospective studies are needed to determine whether these coping dimensions contribute to the development, maintenance, or reduction of suicide risk over time.

## Availability of Data and Materials

For confidentiality reasons, the data from this study are not public. However, access to these data may be obtained by contacting Celia Antuña-Camblor (celia.antuna@udima.es).
