# Supplementary material for: Coping-Strategies as a Mediator Between Symptoms of Sleeping Problems and Risk of Suicide
Source: Actas Esp Psiquiatr. 2026 Oct 15;54(5):1677–87. doi: 10.62641/aep.2384 (PMC13714459; doi:10.62641/aep.2384)
Supplement: Supplementary file 1 [file ActEsp-54-5-1677-1687-s1.zip › AEP-2384-Supplementary materials.docx]

**Supplementary materials**

**Table 1.** Estimated effects of moderated mediation with the sex variable.

|  |  |  | **95% CI** | |
| --- | --- | --- | --- | --- |
| **Independent variable** | **Mediator** | **Moderated mediation index (SE)** | **LL** | **UP** |
|  |  |  |  |  |
| Sleep problems | SSS | -.037 (.059) | -.164 | .077 |
|  | OEE | -.091 (.058) | -.204 | .022 |
|  | REL | .042 (.039) | -.030 | .128 |
|  | PSF | .097 (.070) | -.020 | .259 |
|  | AVO | -.048 (.049) | -.132 | .051 |
|  | NAF | .003 (.031) | -.043 | .070 |
|  | PRE | -.042 (.045) | -.141 | .021 |

*Note*: SSS: Social Support Seeking; OEE: overt emotional expression; REL: Religion; PSF: Problem Solving Focus; AVO: Avoidance; NAF: Negative Auto-Focused; PRE: Positive Reappraisal; LL: lower limit; UL: upper limit.

Given that previous literature has shown a high incidence of the sex variable in the suicide, the mediation model proposed in the text but moderated by the sex variable, have been calculated in a supplementary way to see if they differed as a function of this variable. The results showed that gender was not a significant moderator. The results of the moderated mediation indices are shown in table 1 of the supplementary materials.

In light of these models, it was decided to use the sex variable only as a covariate, as the prevalence data show marked differences, and it is important to control for possible interference with the mediation models proposed.
